# Ego Depletion Does Not Interfere With Working Memory Performance

**DOI:** 10.3389/fpsyg.2018.00538

**Published:** 2018-04-12

**Authors:** Ranjit K. Singh, Anja S. Göritz

**Affiliations:** Department of Psychology, Albert-Ludwigs-Universität Freiburg, Freiburg im Breisgau, Germany

**Keywords:** self-control, ego depletion, working memory, Stroop task, e-crossing task, letter-number sequencing task

## Abstract

Ego depletion happens if exerting self-control reduces a person’s capacity to subsequently control themselves. Previous research has suggested that ego depletion not only interferes with subsequent self-control but also with working memory. However, recent meta-analytical evidence casts doubt onto this. The present study tackles the question if ego depletion does interfere with working memory performance. We induced ego depletion in two ways: using an e-crossing task and using a Stroop task. We then measured working memory performance using the letter-number sequencing task. There was no evidence of ego depletion interfering with working memory performance. Several aspects of our study render this null finding highly robust. We had a large and heterogeneous sample of *N* = 1,385, which provided sufficient power. We deployed established depletion tasks from two task families (e-crossing task and Stroop), thus making it less likely that the null finding is due to a specific depletion paradigm. We derived several performance scores from the working memory task and ran different analyses to maximize the chances of finding an effect. Lastly, we controlled for two potential moderators, the implicit theories about willpower and dispositional self-control capacity, to ensure that a possible effect on working memory is not obscured by an interaction effect. In sum, this experiment strengthens the position that ego depletion works but does not affect working memory performance.

## Introduction

Ego depletion describes a phenomenon in which exerting self-control reduces a person’s capacity to subsequently control themselves (Baumeister et al., 1998). This concept has inspired a large body of work (Hagger et al., 2010). The phenomenon is of great practical importance, providing an explanation of the ebbs and flows of state self-control capacity in everyday life. Moreover, ego depletion is methodologically valuable because it allows for experimental manipulation of self-control capacity, which is crucial for research on the causal effects of self-control in general. An earlier meta-analysis of more than 100 ego depletion studies strongly supported ego depletion (Hagger et al., 2010), and the only disagreements were with regard to the mechanisms underlying ego depletion (Hagger et al., 2010). Recently, however, new meta-analytic evidence (Carter et al., 2015) and replication efforts (Hagger et al., 2016) have cast doubt on whether the ego depletion effect is as stable and universal as previously assumed. While the most recent meta-analysis by Dang (2017) amends the criticism of Carter et al. (2015), the question remains of why the ego depletion effect varies so much across studies.

One explanation for the apparent volatility of ego depletion is that there is no consensus on which tasks and constructs are actually linked to ego depletion. As Carter et al. (2015) noted, several dependent measures for ego depletion have been used in an inconsistent way across studies, such that the reduction of these dependent variables in some studies and their increase in others have been interpreted as evidence for depletion. Furthermore, the ego depletion effect varies considerably in strength depending on which depleting task is being used (Carter et al., 2015; Dang, 2017). Consequently, narrowing down which constructs are tied to ego depletion will help in solving the current controversy surrounding the effect. In our study, we focused on one construct: working memory. It has long been assumed that ego depletion interferes with working memory and that exerting working memory effort induces ego depletion, and some experiments seem to support this assumption (Schmeichel, 2007; Hagger et al., 2010). In addition to these empirical indications, several theoretical arguments speak to a link between working memory and ego depletion: Ego depletion seems to also affect several other executive functions, such as attention control and inhibitory control (Hagger et al., 2010), and thus, it might affect working memory as well. Furthermore, working memory is necessary to uphold goal-oriented self-control, and in turn, self-control should be necessary to defend working memory against distractions (Diamond, 2013). However, the meta-analysis of Carter et al. (2015) draws into doubt, if ego depletion affects working memory performance. They did find an effect of ego depletion on working memory. However, they also found evidence for small-study effects and publication bias. After statistically correcting for these problems, the effect of ego depletion on working memory became non-significant. This suggests that the effect may have been spurious.

In our study, we set out to test whether ego depletion interferes with working memory processes in a highly powered experiment. We recruited a large sample (*N* = 1,935) via the WiSoPanel, a German academic online-access panel (Göritz, 2009, 2014). Using a sequential task paradigm, we induced ego depletion and then measured the participants’ working memory capacity. In a classic sequential task paradigm (Hagger et al., 2010), participants first complete a task that has two versions. Some participants become ego-depleted by completing a version of the first task that is self-control intense. The other participants complete a version of the first task that is markedly less self-control intense, but otherwise as similar as possible to the depleting version. Next, all participants complete a second task that requires self-control but is otherwise dissimilar to the first task. Ego depletion manifests in a reduced performance in the second task if participants completed the depleting version of the first task. For example, Baumeister et al. (1998) used a sequential task paradigm, in which participants observed an emotional video clip. The first task was to watch the video clip without showing any outward signs of emotion (self-control intense condition) or to let their emotions flow freely while watching (less self-control intense). As a second task, participants were asked to solve anagrams, which was the subsequent performance measure.

To induce ego depletion, we chose to adapt the classic sequential task paradigm. Instead of just two task versions, one depleting and one non-depleting, we deployed more task versions to ensure that a potential null finding could not merely be attributed to the specific operationalization. Drawing from meta-analytic evidence (Hagger et al., 2010), we chose two different kinds of depletion tasks: e-crossing tasks and Stroop tasks. For each of these task types, we deployed a non-depleting control version and two different depleting versions. In total, we used six task versions to either deplete or not deplete the participants. Both task types (e-crossing and Stroop) are often used in ego depletion research and have significantly induced depletion in prior studies (Dang, 2017). The specific web-based tasks used in our study are based on the tasks Singh and Göritz (unpublished) developed to induce and measure ego depletion online. They successfully induced ego depletion using the e-crossing task in two online experiments (*N* = 122; *N* = 788). Ego depletion was measured as performance in a subsequent color Stroop task. At the time of writing, the manuscript is still in revision. The manuscript can be obtained upon request from the corresponding author. In the following, we give an overview of the tasks that we used, while the details have been reserved for the section “Materials and Methods.”

Three conditions in our study were based on the e-crossing task. The e-crossing paradigm has been frequently used to induce ego depletion (Hagger et al., 2010). Even though a replication effort cast doubt on the task’s effectiveness (Hagger et al., 2016), the task’s effectiveness is supported by recent meta-analytical findings (Dang, 2017) and by a high-powered study with an e-crossing task adaption that is closer to the original e-crossing task (Singh and Göritz, unpublished) than, for example, Hagger et al. (2016).

The classic e-crossing task is a stimulus detection task with two rounds. In the non-depletion version, participants cross out all Es in two subsequent texts. Our e-crossing non-depletion condition follows this approach as well. In the depletion condition, participants likewise cross out all Es in the first phase, but in the second phase, they have to follow effortful rules on which Es to cross out and which Es to leave unmarked. The drawback of this approach is that the first half of the task is identical between the non-depletion and depletion conditions. Consequently, only half of the task time is spent on inducing ego depletion. In the study at hand, we addressed this drawback by implementing two variations of the depletion condition to intensify the self-control demands of the task. In the single-phase condition, we asked participants to follow effortful rules for the whole task duration, rather than just half of it. In the rule-switch condition, participants followed the same effortful rules in the first half of the task and then had to follow a new rule in the second phase that ran counter to the previous rules.

Three conditions in our study were based on a Stroop task. The Stroop task is a frequently used method of depletion (Hagger et al., 2010; Dang, 2017). Our study adapted a web-based color naming Stroop task by Singh and Göritz (unpublished). The Stroop effect occurs if people see a color word displayed in another color (e.g., “GREEN” displayed in yellow) and are asked to indicate the display color instead of the word meaning (Macleod, 1991). Participants need to inhibit their habitual tendency to indicate the word meaning instead of the display color. Stroop tasks thus draw on a central aspect of self-control: inhibitory control (Diamond, 2013). If the word meaning and the display color match, however, no inhibition is necessary. Using Stroop tasks to induce ego depletion makes use of this by demanding inhibition in the depletion condition and no inhibition in the non-depletion condition. In our Stroop non-depletion condition, the participants completed a Stroop task in which the display color and word meaning always matched and no inhibition was necessary. As with the e-crossing task, we strove to intensify the depletion potential of the Stroop task by devising two strenuous adaptions. In the Stroop exception condition, we asked participants to complete a Stroop task while following an additional rule that runs counter to the usual Stroop instruction: whenever the word is displayed in red, participants should indicate the word meaning. In the Stroop two-phases condition, the participants followed the customary Stroop instructions (indicating the display color) for half of the task, and then had to follow an incompatible rule in the second half (indicate a color that matches neither the word meaning nor the display color).

To measure the working memory capacity of the participants after the depletion tasks, we devised a web-based adaption of the letter-number sequencing task from the German adaption of the Wechsler Adult Intelligence Scale III (von Aster et al., 2006). This letter-number-sequencing task has the advantage that it not only relies on keeping information in short term memory but also requires actively modifying the information and thus taxing working memory (Diamond, 2013). In our web-based version, the participants observed sequences of numbers and letters that they needed to memorize and then reorder the numbers in ascending order and the letters in ascending order, and then, they finally type the reordered sequences in a text field. During the task, the participants worked on increasingly longer sequences. In the original task, performance was scored by counting the number of correct trials. Our task adaption computes this classic performance measure but also aggregates several more finely grained measures as detailed in the methods section. By computing a wide range of task metrics, we aimed to ensure the best possible chance of detecting an effect of ego depletion on working memory performance.

To increase our ability to detect the ego depletion effect, we measured two constructs to control for them as potential covariates and moderators: participants’ dispositional self-control capacity and participants’ implicit theories about willpower (ITWP).

Trait self-control describes the way in which people vary in their average self-control success across time and different situations (de Ridder et al., 2012). Including dispositional self-control capacity may increase the power of our study in two ways. First, as a covariate, a measure of dispositional self-control capacity controls for some of the interindividual variance of self-control capacity that participants bring into our experiment. Second, dispositional self-control capacity might act as a moderator if people with high self-control capacity are more resilient to ego depletion. It is conceivable that people who draw from a large self-control capacity pool can more easily compensate efforts and are less affected by depletion.

Implicit theories about willpower are another promising moderator of ego depletion. The ITWP are assumptions people hold about how exerting self-control affects themselves. ITWP span a continuum between people who assume that their willpower is unlimited and people who assume that their willpower is highly limited. Those who believe their willpower to be unlimited assume that exerting self-control effort has no effect on them (Job et al., 2010). Relevant for the present study is that these beliefs interact with the ego depletion effect (Job et al., 2010; Savani and Job, 2017; Singh and Göritz, unpublished). Consequently, including the ITWP as a moderator increases power and thus the ability to detect ego depletion (Savani and Job, 2017; Singh and Göritz, unpublished).

In summary, we set out to test if ego depletion affects working memory capacity or not. Consequently, we tried to ensure that a potential null finding is informative. To this end, we recruited a large sample to ensure a high-powered experiment. We also spread the risk of a specific ego depletion paradigm failing to induce ego depletion by relying on several ego depletion paradigms. To measure working memory capacity reliably, we adapted an established measure as faithfully as possible for the web. We also generated several alternative performance scores to make the task more sensitive to small changes in working memory capacity. Lastly, we introduced two important control variables, dispositional self-control capacity and ITWP, to better isolate depletion.

## Materials and Methods

### Ethics Statement

We conducted this study in accordance with the German Psychological Society’s (DGPs) ethical guidelines, as well as the APA’s ethical standards. According to the German Psychological Society’s ethics commission, approval from an institutional research board needs to be obtained only if funding is subject to ethical approval by an Institutional Review Board. This research was reviewed and approved by the German Science Foundation (Grant Number GO 1107/14-1), which did not require additional Institutional Review Board approval. The participants volunteered and received the standard reward of 1.5 € in the WiSoPanel. The participants were aware of taking part in the research. They were informed of what to expect in the study, and they provided informed consent. Participants not willing to provide informed consent were free to drop out of the study immediately and still receive payment. All participants provided informed consent. All data were collected and analyzed anonymously.

### Participants

We recruited 1,935 participants via the WiSoPanel, a German academic online-access panel (Göritz, 2009, 2014). Of those, 1,434 (74%) participants completed the whole study. The average age was 49.8 years (*SD* = 14.3). Of the participants, 57.5% were women, and 32.3% had a university degree. The majority of participants were working (59.4%), and 10.9% were students. Only 5% of the participants were psychologists or were studying psychology.

We excluded 66 participants from our analysis because they used a smartphone despite being instructed not to. We felt that smartphones might be used in contexts not appropriate for a concentration-intense online experiment. We also cautiously excluded some participants based on their (lack of) performance in the depletion task. We excluded participants based on percentiles: We ranked all participants who completed the Stroop task in each condition based on the number of errors they made. We then excluded the 1% of participants (8) with the most errors in each condition. Similarly, we ranked all participants who completed the e-crossing task by the number of correct Es they identified. We then excluded the 1% of participants (8) who clicked the fewest correct Es. In total, we excluded 82 participants.

The analyses were based on *N* = 1,385 participants. Please note that this is slightly higher than the number of completed surveys (1,434) minus the 82 excluded participants. This is because a few participants completed everything except the debriefing page. The randomization was successful: The number of participants who completed the respective depletion tasks did not vary between the six conditions, *X*^*2*^(5, *N* = 1,791) = 4.31, *p* = 0.506.

### Procedure

After giving informed consent, participants were asked whether they were studying or had studied psychology. Participants then filled out the 13 items of the German short version of the Self-Control scale (SCS-K-D, Bertrams and Dickhäuser, 2009) and six items about their ITWP (Job et al., 2010). Next, participants were randomly assigned to one of the six conditions: e-crossing non-depletion (*n* = 289), e-crossing single-phase (*n* = 274), e-crossing rule-switch (*n* = 310), Stroop non-depletion (*n* = 295), Stroop exception (*n* = 304), and Stroop two-phases (*n* = 319). (For the number of participants in each condition that were part of the final analyses, see **Table 2**.) After completing the respective task, the participants were asked about their current mood and how they had experienced their task. Next, the participants completed the letter-number sequencing task, which served as a dependent measure. After the task, the participants were again asked about their current mood and how they had experienced the letter-number sequencing task. Lastly, participants were asked which device and input method they were using.

### Materials

#### Implicit Theories About Willpower

We included the German versions of six items that capture a participant’s implicit theories about “willpower” (in the sense of self-control) in the domain of mentally effortful tasks (Job et al., 2010). These items address the question of whether participants think that their willpower can be depleted, or if they think that their willpower is unlimited. These items were included as a potential moderator of the ego depletion effect based on research by Job et al. (2010), who found that the belief in unlimited willpower mitigated ego depletion. The six items were balanced in that three postulated a depletable willpower and three postulated an unlimited willpower. This was to ensure that the items did not prime the participants one way or another. An example item was: “After you have completed a difficult task, you are not able to continue with something new with the same attention. You have to recover first.” The participants responded on a six-point Likert scale with the endpoints “totally disagree” and “agree strongly.” The internal consistency of the ITWP scale was Cronbach’s α = 0.82.

#### Self-Control Scale

We included the German short version of the self-control scale (SCS-K-D, Bertrams and Dickhäuser, 2009) to measure the participants’ dispositional self-control capacity. The German translation is based on the original (English) self-control scale by Tangney et al. (2004). Dispositional self-control capacity may act as a moderator of ego depletion in that people with high self-control capacity have more leeway to mitigate ego depletion, while people with low self-control capacity may be hard-pressed to compensate for prior effort. The SCS-K-D consists of 13 items, such as “I am able to work effectively toward long-term goals.” Nine of the items are reversed in that the high answers reflect low dispositional self-control. For example, “I say inappropriate things.” The participants responded on a six-point Likert scale with the endpoints “doesn’t apply at all” and “absolutely applies.” The internal consistency of the SCS-K-D in our sample was Cronbach’s α = 0.84.

#### Self-Assessment Manikin

The Self-Assessment Manikin (SAM) is a pictorial assessment technique that measures participants’ affective reaction to stimuli (Bradley and Lang, 1994). We chose the SAM as an economical and easy-to-understand measure. We used the valence scale of the SAM to assess participants’ mood after the e-crossing task. Participants indicated how they felt by choosing an abstract representation of a person who is frowning deeply, frowning slightly, looking neutral, smiling slightly, or smiling broadly. We added the scale anchors “unhappy” and “happy” to the outermost pictograms to make it self-explanatory.

#### Task Evaluation Questions

The participants answered four questions regarding how they had experienced the task after the depletion task and after the letter-number sequencing task. The participants were asked (1) how easy it was to understand the task (hard to understand—easy to understand), (2) how varied the task was (monotonous—varied), (3) how pleasant the task was (unpleasant—pleasant), and (4) how effortful the task was (effortful—easy). Each question was answered on a five-point scale.

#### E-Crossing Task

We developed a web-based adaption of the e-crossing task, which has frequently been used to deplete participants (Hagger et al., 2010). The task is a stimulus detection task. The participants observed a long text in which they were asked to find and click on lowercase and uppercase Es. The texts were displayed in a large (22 px) monospace font so that the individual letters were easy to click on. Clicking on an E marked that E with a green background so that participants had visual feedback. Clicking a marked E unmarked the E. The task differed depending on the three e-crossing conditions: a non-depletion condition and two depleting conditions: single-phase and rule-switch. In the non-depletion condition, the task consisted of two phases that lasted three minutes each. Participants were shown a different text in each round. They were asked to click on all Es in the texts.

In classic implementations of the e-crossing task, the first phase is identical between non-depletion and depletion. Only in the second depletion phase, participants have to follow effortful rules. Consequently, only half of the task time is used for depletion. With our two e-crossing depletion conditions, we strove to use the whole task time for depletion.

In the single-phase condition, participants completed one 3-min long phase, which was as long as the two phases of the non-depletion condition together. Participants were asked to click on Es in the text while following two rules: (1) Do not click on Es that are followed by a vowel (e.g., reign) and (2) Do not click on Es that are two letters away from a vowel (e.g., forest). This task version uses the whole task time to place self-control demands on the participants. However, unlike in the classic depletion paradigm, this approach entails that participants do not need to get used to one task instruction (click on all Es), and then inhibit this established pattern in the second phase (don’t click on Es that fall under the two rules).

In the rule-switch condition, we allowed for the possibility that establishing and then counteracting a behavioral pattern is a crucial component of the e-crossing task. In this condition, participants completed two phases, each 3 min long. The first phase used the same instructions as the single-phase condition: The participants were asked to click on Es in the text while following the two rules. In the second phase, the participants were again asked to click on Es in the text, but this time only to click on Es that were two letters away from a vowel. With these instructions, the participants needed to click on Es that were previously excluded by the second rule. At the same time, they had to refrain from clicking on Es that were correct in the first phase.

#### Color Stroop

We developed a modified color Stroop task. Stimuli were color words (RED, GREEN, BLUE, YELLOW) displayed in one of these four colors. The task consisted of 100 trials with an equal distribution of colors. Each word was displayed until the participants responded. Next, feedback on whether the answer was correct was displayed for 500 ms, followed by the next word. The participants responded by clicking with the mouse on one of four buttons below the stimulus area. The buttons were labeled “red,” “green,” “blue,” or “yellow” in black text color. The button order was randomized for each participant, and the buttons were displayed in a two by two grid. This allowed participants to rest their mouse cursor in the center with all buttons at the same distance.

The task differed depending on the three color Stroop conditions: a non-depletion condition, a depleting condition “exception” and another “two-phases” depleting condition. In the Stroop non-depletion condition, participants were asked to indicate the displayed color of a word, while ignoring the word meaning. All stimuli were congruent, meaning that the word meaning and displayed color matched (e.g., “GREEN” displayed in green). In the two Stroop depletion conditions, we strove to intensify the ego depletion induction with two different approaches.

In the Stroop exception condition, all stimuli were incongruent, meaning that the word meaning and displayed color did not match (e.g., “BLUE” displayed in yellow). The participants were asked to indicate the displayed color of a word, while ignoring the word meaning, but with one exception: If the displayed color was red, they were asked to indicate the word meaning. If, for example, “BLUE” was displayed in yellow, the correct answer was “yellow.” If, however, “BLUE” was displayed in red, the correct answer was “blue.” The exception rule increases self-control demands via two mechanisms. First, the participants needed to adhere to a second rule, which introduces a parallel self-control demand. Second, the participants are forced to switch tasks on a trial basis, from indicating display color to indicating word meaning.

In the Stroop two-phases condition, the 100 trials were split into two phases of 50 trials each. All stimuli were incongruent. In the first phase, the participants were asked to indicate the displayed color of a word, while ignoring the word meaning. In the second phase, the participants were asked to click on a color button that neither corresponded to the display color nor with the word meaning. If, for example, “BLUE” was displayed in yellow, correct answers were “red” or “green.” This new rule increased depletion because the participants still need to inhibit their habitual tendency to indicate the word meaning, but now also need to inhibit the behavioral pattern established in the first phase of clicking on the displayed color.

It should be noted that many prior ego depletion studies using the Stroop task only used a Stroop task version with all incongruent trials to deplete participants. Our two depleting Stroop versions retain this basic characteristic: Participants still have to overcome the classic interference between word meaning (distractor) and display color (target). Our modifications merely add an additional challenge to this, while retaining the original self-control demand: The exception condition adds a second, parallel rule and the two-phases condition adds a rule change midway.

#### Letter-Number Sequencing Task

To measure the current working memory capacity of the participants, we adapted a task from the Wechsler Adult Intelligence Scale III (von Aster et al., 2006): the letter-number sequencing task. In our adaption, the participants were instructed to pay attention to the sequences of letters and numbers they were about to see. Their task was to remember the current sequence, reorder it in their head, and then to type the reordered sequence into a text input field. The rule for reordering was to start listing all numbers in ascending order, and then all letters in ascending order. For example, the sequence “4G82H” would become “248GH.” The participants completed 21 rounds in total. The rounds started with sequences of two characters and added a new character after every third round. The longest sequences were eight characters long.

In each round, the participants observed a fixation dot for 1,000 ms. Next, a sequence of letters and numbers was displayed one character at a time. Each character was displayed for 500 ms, followed by no character for 750 ms, which was then followed by the next character. After the whole sequence had been displayed, a text input field appeared and below it a “save answer” button. The answer field was put into focus automatically, which meant that it did not needed to be clicked and that on mobile devices, the on-screen keyboard appeared automatically. After writing down the reordered sequence, the participants could submit it with the Enter key or by clicking or tapping the “save answer” button. The text field did not allow typed characters to be deleted again. This prevented participants typing the original sequence before reordering it to relieve their short-term memory.

We created a number of aggregated measures to capture the potential ego depletion effect on working memory in a comprehensive, yet precise manner. We formed several performance scores, time-based scores, and some control measures.

We used the following performance scores: (1) We aggregated the classic score used in the original letter-number sequencing task, counting the number of correct trials (a correct trial is a completely recalled and correctly reordered sequence of letters and numbers). (2) We created a score representing the longest correct trial. (3) We created a score that was the sum of all characters of the correct trials. This is similar to the classic score, except that now the trials were weighted by their length; a trial with a sequence of two characters, for example, would net two points, a sequence of five characters, five, and so on. (4) We computed a less strict score by finding the longest correct subsequence in every answer. For example, if the correct sequence was “248GH” and a participant answered only partially (e.g., “248G”) then the longest correct subsequence would be four. This algorithm disregarded additional characters: In the example, the answer “1248GJ” would also net four points due to the correct subsequence “248G.” This score is highly, but not perfectly, correlated with the third score, which was the sum of all the characters of the correct trials, because the longest subsequence in a correct trial is the number of characters of that trial. The two scores deviate for participants who sometimes gave partially correct answers. Consequently, it is finer grained. (5) We measured the longest correct subsequence across all trials, which was similar to the second score.

Because depletion might affect the speed with which the participants complete the task, we aggregated two time-based measures: (1) We created a score of the average time per trial in milliseconds. (2) We created a score representing the time of the slowest trial in milliseconds, in case the ego depletion manifested in long but infrequent lapses in attention.

Lastly, we created three control measures that gave more insight into the answering behavior of the participants: (1) We created a score representing the number of trials in which participants correctly recalled the sequence but failed to reorder it. This might indicate a lapse in the self-control necessary to closely follow instructions. (2) We created a score of the sum of the correctly recalled characters, regardless of order. This score was intended to reflect the short-term memory component of the task (simple recall), as opposed to the working memory component (modifying the content of the short-term memory). (3) We created a score of the number of trials in which the participants gave no answer at all.

## Results

Before testing the hypothesis, we examined the three tasks used in our experiment: the e-crossing task, the Stroop task, and the letter-number sequencing task. Since many task outcomes are necessarily asymmetrically distributed (e.g., error distributions), we use the median (*Mdn*) and the interquartile range (*IQR*) instead of the arithmetic mean and *SD*. The Median and *IQR* are robust against outliers and do not require symmetrical distributions.

In the e-crossing task conditions, the participants engaged well with the task. In the e-crossing non-depletion condition, participants clicked on *Mdn* = 123 (*IQR* = 35) Es in the first round. This level of effort was maintained in the second round with *Mdn* = 124 (*IQR* = 40.25) clicked Es. In the two e-crossing depletion conditions, fewer Es were clicked because of the rules restricting which Es were correct. The participants in the e-crossing single-phase condition clicked *Mdn* = 69 Es (*IQR* = 40), of which *Mdn* = 55 (*IQR* = 30) Es were correct. In the e-crossing rule-switch condition, the participants clicked on *Mdn* = 38 (*IQR* = 24) Es in the first phase, of which *Mdn* = 30 (*IQR* = 19) were correct. This level of effort is similar to the two experiments by Singh and Göritz (unpublished), in which the web-based e-crossing task adaption was validated and shown to induce ego depletion.

In the Stroop task conditions, the participants worked diligently. Most participants made few or no mistakes at all. In the Stroop non-depletion condition, the participants made *Mdn* = 0 (*IQR* = 1) mistakes. In the Stroop exception condition, the participants made *Mdn* = 2 (*IQR* = 5) mistakes in 75 inconsistent trials, and *Mdn* = 1 (*IQR* = 6) mistakes in the 25 exception trials. In the Stroop two-phases condition, participants made *Mdn* = 0 (*IQR* = 1) mistakes in the first phase and *Mdn* = 1 (*IQR* = 3) mistakes in the second phase. Meanwhile, the median reaction times replicate the classic Stroop effect, demonstrating that the depletion conditions required more effort than the non-depletion conditions. We compared the congruent trials in the non-depletion condition to the incongruent trials in the first phase of the two-phase condition and the incongruent trials of the exception condition. Reaction times in the three Stroop conditions differed, *F*(2,871) = 222.68, *p* < 0.001, $\eta_{p}^{2}$ = 0.338. *Post hoc* tests using the Sheffé correction showed that the inconsistent trials in both depletion conditions were answered more slowly than the congruent trials in the control condition (*p* < 0.001 for both comparisons). Furthermore, the *post hoc* tests showed that the incongruent trials in the exception condition were even slower than the incongruent trials in the two-phases condition (*p* < 0.001).

As a manipulation check, we tested if the depletion conditions were more effortful than the non-depletion conditions. The participants rated the depletion task on a five-point scale on whether it was effortful or easy. Participants’ perception of effort varied among the Stroop task versions, *F*(2,842) = 93.09, *p* < 0.001, $\eta_{p}^{2}$ = 0.181. Contrasts revealed that both depleting Stroop task versions were perceived to be more effortful than the non-depletion Stroop task version. The Stroop exception version was rated 1.3 scale points more effortful than the Stroop non-depletion version (*SE* = 0.10, *p* < 0.001). The Stroop two-phases version was rated 0.97 scale points more effortful than the Stroop non-depletion version (*SE* = 0.10, *p* < 0.001).

The same pattern emerged in the e-crossing task. The participants’ perception of effort varied among the e-crossing task versions, *F*(2,719) = 50.84, *p* < 0.001, $\eta_{p}^{2}$ = 0.124. Contrasts revealed that both depleting e-crossing task versions were perceived to be more effortful than the non-depletion e-crossing task version. The e-crossing single-phase version was rated 0.82 scale points more effortful than the e-crossing non-depletion version (*SE* = 0.11, *p* < 0.001). The e-crossing rule-switch version was rated 0.95 scale points more effortful than the e-crossing non-depletion version (*SE* = 0.10, *p* < 0.001). In **Table 1**, we have summarized the mean and standard deviation for all four task evaluation questions for all conditions.

**Table 1:** Mean and (*SD*) values for the task evaluation questions.

	Hard to understand—Easy to understand	Effortful — Easy	Monotonous — Varied	Unpleasant — Pleasant
Stroop non-depletion	4.77 (0.63)	4.10 (1.17)	2.22 (1.35)	3.28 (1.14)
Stroop exception	3.73 (1.15)	2.85 (1.10)	2.74 (1.22)	3.19 (1.07)
Stroop two-phases	4.43 (0.91)	3.13 (1.16)	3.32 (1.17)	3.56 (1.09)
e-crossing non-depletion	4.66 (0.94)	3.67 (1.20)	2.22 (1.28)	3.22 (1.16)
e-crossing single-phase	3.62 (1.19)	2.85 (1.18)	2.40 (1.11)	3.04 (1.10)
e-crossing rule-switch	3.59 (1.24)	2.72 (1.06)	2.82 (1.19)	3.16 (1.09)

In our dependent measure, the letter-number sequencing task, the participants showed good effort. Nearly no participant shirked the task by giving no answers, *Mdn* = 0 (*IQR* = 0). Similarly, almost no one misunderstood the instructions and merely repeated the sequence in its original order, *Mdn* = 0 (*IQR* = 0). The participants answered *Mdn* = 12 (*IQR* = 5) trials of 21 trials correctly. Our depletion tasks did not cause systematic dropout. The number of people who completed the letter-number sequencing task did not vary by depletion task condition, *X*^*2*^(5, *N* = 1434) = 6.19, *p* = 0.288.

With regard to our central research question — does ego depletion interfere with working memory — we performed several analyses. Because we calculated several scores from the letter-number sequencing task, we began with a MANOVA that included all possible dependent measures. Next, we performed a MANOVA with only the direct performance measures because including fewer dependent variables increases power. We then calculated an ANOVA using the classic letter-number sequencing performance score (number of correct trials) as the dependent variable. It is possible, however, that one of our alternative performance scores is more sensitive to ego depletion than the classic score. Consequently, we also performed an ANOVA using the performance score with the lowest *p*-value in the MANOVA and thus the highest chance of reaching significance. Next, we looked at a *T*-test comparing performance in the two non-depletion conditions with performance in the four depletion conditions. Lastly, we ran models that incorporated either the ITWP or the dispositional self-control capacity as a moderator.

Based on the meta-analysis by Carter et al. (2015), a null finding is a likely outcome. Consequently, we present a wide range of detailed analytic approaches to maximize our chance of finding an effect if it exists. In other words, we accept some degree of type-1 error cumulation to increase power, thus rendering a potential null finding more robust.

To test if our depletion task conditions influenced performance in the letter-number sequencing task, we ran a MANOVA with a factor including all six depletion task conditions. As dependent variables, we included all scores derived from the letter-number task, as is detailed in the method section. There were five performance scores, two time-based measures, and three measures of participant behavior. In **Table 2**, we have summarized the means and standard deviation for all scores derived from the letter-number task. The *n* for each condition represents the number of participants in each condition that were part of the analyses. The depletion task conditions did not significantly influence the dependent measures, *F*(50,6251.53) = 0.936, *p* = 0.602, *Wilk’s* Λ = 0.967, $\eta_{p}^{2}$ = 0.007.

**Table 2:** Mean and (*SD*) of all measures derived from the letter-number sequencing task for all experimental conditions.

	Stroop conditions			e-crossing conditions		
	Non-depletion	Exception	Two-phases	Non-depletion	Single-phase	Rule-switch
	(*n* = 242)	(*n* = 226)	(*n* = 254)	(*n* = 232)	(*n* = 200)	(*n* = 231)
**Performance scores**						
Number of correct trials	11.63 (4.54)	11.32 (4.37)	11.46 (4.52)	11.47 (4.44)	11.92 (4.00)	10.87 (4.65)
Length of the longest correct trial	6.17 (1.77)	5.96 (1.68)	6.07 (1.84)	6.02 (1.82)	6.25 (1.51)	5.90 (1.82)
Sum of all characters of the correct trials	47.20 (23.77)	44.98 (23.07)	46.19 (23.87)	45.85 (23.34)	47.66 (21.43)	43.19 (23.50)
Sum of all correct subsequences	70.36 (19.62)	67.40 (20.11)	69.17 (20.20)	69.41 (20.28)	70.64 (17.93)	65.99 (21.54)
Length of the longest correct subsequence	6.27 (1.60)	6.07 (1.57)	6.21 (1.58)	6.16 (1.59)	6.35 (1.38)	6.02 (1.69)
**Time based scores**						
Average time per trial (ms)	11719 (22786)	10354 (7690)	9739 (4967)	10264 (11553)	11103 (14434)	10443 (7093)
Time of the slowest trial (ms)	65827 (416706)	38996 (117415)	31362 (48187)	43585 (218267)	52047 (290030)	34645 (85784)
**Answering behavior**						
Number of trials with correct recall but original order	0.36 (1.12)	0.26 (1.00)	0.22 (0.80)	0.29 (1.03)	0.17 (0.71)	0.40 (1.53)
Sum of correctly recalled characters, regardless of order	84.30 (16.09)	81.45 (18.28)	81.91 (19.20)	83.03 (18.19)	83.85 (17.32)	80.29 (20.31)
Number of trials with no answer	0.50 (1.48)	0.70 (1.86)	0.68 (1.92)	0.72 (2.22)	0.76 (2.29)	0.84 (2.50)

We ran a MANOVA with only a selection of four promising performance scores: (1) the number of correctly answered trials, (2) the sum of the characters of all correct trials, (3) the sum of the longest correct subsequence of each trial answer, and (4) the sum of all recognized characters regardless of order. Again, the depletion task conditions did not significantly influence the dependent measures, *F*(20,4564.63) = 1.296, *p* = 0.169, *Wilk’s* Λ = 0.981, $\eta_{p}^{2}$ = 0.005.

We ran an ANOVA using only the performance score used in the original letter-number sequencing task: the number of correctly answered trials. Again, the depletion task condition did not significantly affect the working memory performance measure, *F*(5,1379) = 26.27, *p* = 0.247, $\eta_{p}^{2}$ = 0.005.

We ran a second ANOVA using the performance score that most closely approached significance in the MANOVAs: the sum of the longest correct subsequence of each trial answer. Again, the depletion task condition did not significantly affect the working memory performance measure, *F*(5,1379) = 1.82, *p* = 0.106, $\eta_{p}^{2}$ = 0.007.

In an attempt to make the most of our sample size, we combined the six depletion task versions to test the two non-depletion conditions against the four depletion conditions. As the dependent measure, we used the empirically most promising performance score: the sum of the longest correct subsequence of each trial answer. However, the score did not differ significantly between the non-depletion conditions (*M* = 69.90, *SD* = 19.93) and the depletion conditions (*M* = 68.25, *SD* = 20.10), *t*(1383) = 1.45, *p* = 0.147.

We controlled for potential moderators of the ego depletion effect to try to isolate a significant effect of depletion on working memory performance. As moderators, we tested ITWP and dispositional self-control capacity. Because both moderators are continuous, we estimated regression models using PROCESS (Hayes, 2013). We standardized all variables, except the dichotomous depletion factor, to arrive at standardized coefficients (β). All of our PROCESS models used the HC3 heteroscedasticity-consistent standard error estimator, as Hayes and Cai (2007) recommend for all OLS regressions. We used an indicator coding scheme to include our categorical depletion task conditions into the regression. Consequently, we ran the three Stroop conditions and the three e-crossing conditions separately. In each, we compared the two depletion task versions with the non-depletion version as a reference category. Including the ITWP as a moderator did not significantly improve the Stroop regression model (Δ*R*^2^ < 0.001, *p* = 0.837), nor the e-crossing task regression model (Δ*R*^2^ = 0.002, *p* = 0.412). Similarly, including the dispositional self-control capacity as a moderator also did not significantly improve the Stroop regression model (Δ*R*^2^ < 0.001, *p* = 0.916), nor the e-crossing task regression model (Δ*R*^2^ = 0.003, *p* = 0.423). These non-significant interactions showed that the regressions do not add value to the ANOVAs. Thus, working memory performance was not affected by depletion in any of the regression models.

In the spirit of transparency and in light of the current discussion in ego depletion research we have published the dataset underlying this study. You can retrieve it from https://osf.io/mksha/ (Singh and Göritz, 2018). If you intend to analyze the data, please do not hesitate to contact the corresponding author. We will walk you through the dataset and answer any questions you may have.

## Discussion

In our study, we set out to test if ego depletion interferes with working memory performance. To this end, we conducted an online experiment. We induced ego depletion using an e-crossing task and a Stroop task. We measured working memory performance using the letter-number sequencing task. In line with Carter et al. (2015) meta-analytical findings, we found no evidence that ego depletion interferes with working memory performance. This runs counter to earlier findings that suggested such an interference (Schmeichel, 2007; Hagger et al., 2010).

Several aspects of this study’s design make this null finding highly robust. First, the large sample size of 1,385 participants in our analyses. To gauge whether our sample was large enough to consider its null finding stable, we conducted a *post hoc* power analysis. There are different estimates on how large ego depletion effects are. Hagger et al. (2010) estimated an effect size of *d* = 0.62. Dang (2017) estimated an effect of *g* = 0.58 for depletion with crossing out letters tasks (such as the e-crossing task) and an effect of *g* = 0.44 for depletion with Stroop tasks.Carter et al. (2015) arrived at an estimate of *g* = 0.24 for studies using a Stroop paradigm to deplete participants. Basing our power analysis on the most conservative estimate by Carter et al. (2015), the study at hand was highly powered: A comparison of the means of two groups with 911 (depletion conditions) and 474 participants (non-depletion conditions), revealed a power of 0.983, given an effect size of *g* = 0.24 (Faul et al., 2009). In other words, the beta error is smaller than 2%.

Second, we used depletion tasks from two task families (Stroop tasks and e-crossing tasks), thus making it less likely that the null finding is due to a specific depletion paradigm. Both tasks are frequently used in ego depletion research (Dang, 2017). The adaptions of the Stroop task and the e-crossing task for web-based research that our experiment was based upon have been successfully used before (Singh and Göritz, unpublished). Paradata on participant behavior shows that participants performed diligently in both tasks. The Stroop task allowed us to demonstrate that the two depletion conditions were more effortful than the non-depletion condition. Reaction times as a performance measure were significantly and substantially higher in the depletion conditions than in the non-depletion condition. Moreover, the participants experienced the depleting versions of the e-crossing task and the Stroop task as more effortful than the non-depleting versions. This supports the idea that our depletion conditions did indeed induce ego depletion.

Third, to avoid one-sidedness, we derived a number of scores and measurements from our dependent task, the letter-number sequencing task. The reasoning behind this was that ego depletion might manifest differently in different aspects of the working memory task, for which we wanted to be prepared. As such, we calculated performance scores based on correct answers, time-based scores, and scores based on possible mistakes (such as forgetting to reorder the sequence). However, not even *post hoc* cherry-picking the empirically most promising score revealed a significant ego depletion effect on working memory performance.

Fourth, to maximize the chances of revealing an effect, we conducted several different analyses. We ran analyses using several of the scores and measurements from our dependent task. Furthermore, we ran analyses focusing on the score established in the literature (number of correct trials) and the most empirically promising score (the sum of the longest correct subsequence of each trial answer). To make the most of our large sample, we aggregated all depletion conditions into one group and the two non-depletion conditions into another group. However, even the resulting high-powered *t*-test did not show a significant finding.

Fifth, we tested two potential moderators: ITWP and dispositional self-control capacity. The reasoning behind this was that the ego depletion effect might be obscured by an interaction effect. Including moderators can help isolate the ego depletion effect (Singh and Göritz, unpublished). However, both ITWP and dispositional self-control capacity did not interact with ego depletion. Consequently, even with those constructs included in the analyses, no ego depletion effect on working memory performance emerged.

Our study is subject to some limitations. Our study showed that ego depletion did not affect working memory performance. The question remains if the inverse is also true: that working memory effort does not induce ego depletion. Since working memory does not react to ego depletion, it does not seem to rely strongly on self-control. Consequently, it may be that working memory effort does not lead to ego depletion. This assumption is supported by the most recent meta-analysis (Dang, 2017) in which working memory effort did not significantly induce ego depletion. However, our study did not test this and Dang’s (2017) findings are based on only six unpublished studies. Consequently, there is a need for further research. Whether working memory does induce ego depletion needs to be determined in another highly powered experiment. A second point is that we assessed working memory performance in a controlled and abstract manner. This has the advantage of being able to isolate working memory from neighboring constructs, such as attentional control. However, in real-life settings, ego depletion might reduce working memory performance indirectly. Ego depletion could, for example, limit a person’s ability to protect their working memory from external distraction (Diamond, 2013). Future studies could explore this possibility using dual task paradigms. Traditionally, dual task paradigms employ two parallel, interfering tasks. Performance in the secondary task is used as an indicator for the cognitive requirements in the primary task (Power, 1986). This setup might more closely reflect everyday challenges to working memory, as it puts participants under time pressure (the tasks are reaction time tasks), and it features competing demands on a common resource.

Despite these limitations, our study contributed to answering the question of whether ego depletion interferes with working memory. It corroborates the position that working memory processes are not affected by ego depletion (Carter et al., 2015), as was previously assumed (Schmeichel, 2007; Hagger et al., 2010). Our findings suggest that working memory should not be used to measure ego depletion. Pending further research, working memory should perhaps also not be used to induce ego depletion, if alternatives are feasible (Dang, 2017). Consequently, we hope our finding helps researchers to avoid dead ends, such as using working memory tasks for ego depletion measurement.

Furthermore, our finding may open up new avenues for research on executive functions. Future research should test which executive functions are linked to ego depletion and which are not. The emerging pattern might deepen our understanding of both executive functions as well as the ego depletion effect. Moreover, our finding implies that an ego depletion paradigm can be used to discern which cognitive tasks solely rely on working memory (those tasks should be unaffected by prior depletion) and which tasks require other executive functions such as inhibitory control (those tasks might be affected by prior depletion).

## Author Contributions

RS: conception and study-design, acquisition, analysis and interpretation of data, drafting and revising of the work, and final approval. AG: conception, acquisition, analysis, and interpretation of data, revising the work, and final approval. Both authors agreed to be accountable for all aspects of the work in ensuring that questions related to the accuracy or integrity of any part of the work are appropriately investigated and resolved.

## Conflict of Interest Statement

The authors declare that the research was conducted in the absence of any commercial or financial relationships that could be construed as a potential conflict of interest.
